# Effect of a Chitosan Coating Enriched with an Olive Leaf Extract on the Characteristics of Pork Burgers

**DOI:** 10.3390/foods12203757

**Published:** 2023-10-12

**Authors:** Ana Isabel Carrapiso, Manuel Pimienta, Lourdes Martín, Vladimiro Cardenia, Ana Isabel Andrés

**Affiliations:** 1 Tecnología de Alimentos, Escuela de Ingenierías Agrarias, Universidad de Extremadura, Ctra de Cáceres s/n, 06007 Badajoz, Spain; mapigon@gmail.com (M.P.); martinlu@unex.es (L.M.); aiandres@unex.es (A.I.A.); 2 Department of Agricultural, Forest and Food Sciences, University of Turin, 10095 Grugliasco, Italy; vladimiro.cardenia@unito.it

**Keywords:** chitosan coating, pork burgers, olive leaf extract, modified atmosphere packaging, meat color, texture, oxidation

## Abstract

Chitosan coatings have been investigated for improving food shelf-life. The addition of an olive leaf extract could enhance its beneficial effect. The aim of this study was to evaluate the effectiveness of an olive leaf extract added to a chitosan coating in delaying deterioration in refrigerated pork burgers without additives packaged under a 40% oxygen and 60% carbon dioxide modified atmosphere. Some general parameters (microbial counts, instrumental color and texture, and lipid and protein oxidation) were measured over the storage of pork burgers without coating (Control), with a chitosan-based coating (Chitosan) and with a chitosan-based coating enriched with an olive leaf extract (Chitoex). The coating impacted the effect of the storage time on most parameters. Both coatings were especially effective at limiting the changes that occur over time in the headspace gases, some texture parameters (hardness, gumminess, and chewiness) and lipid oxidation, although the effect on the microbial counts was weak. Chitoex was more effective than Chitosan at preventing changes in the headspace gases on day 11 and in lipid oxidation on all the sampling days. In conclusion, the Chitoex coating could be useful for prolonging the storage of pork burgers by preventing changes in texture and reducing lipid oxidation.

## 1. Introduction

The quality of meat and meat products is affected by several factors. Among them, microbial growth and oxidation are often involved in deterioration [1,2]. To prevent them and extend shelf-life, several food additives are often used. However, the current attitudes of some consumers are a leading force in the replacement of synthetic food additives with natural alternatives.

Chitosan is a biopolymer, abundant in crustacea, insects and fungi, with antimicrobial and antioxidant activity, as well as the ability to create semipermeable barriers for gases, such as oxygen and carbon dioxide [3,4]. Some recent reviews have focused on the application of chitosan to food [3,5,6,7]. It has been applied to meat and meat products through different strategies, including the production of chitosan-based films [8,9], added and mixed with ground raw meat during food production [10,11], or used as a coating material [4,12]. As a coating, it can be applied to meat without grinding, as well as to ground meat without reaching the inner parts of the product. In addition, its protective effect persists even after the package has been opened.

To increase the antimicrobial and antioxidant effect of chitosan, several studies have proposed the addition of plant extracts, such as anise [13], rosemary [9], basil [10] and lemongrass [14] extracts. Olive leaf extracts have recently drawn some attention because of their antioxidant and antimicrobial properties [15,16,17,18], as well as their health benefits [18,19,20]. In the last decade a number of studies have investigated their components, the most abundant being oleuropein, followed by other less abundant compounds, such as hydroxytyrosol, tyrosol, verbascoside and rutin, whose relative concentration depends on the extraction method and other abiotic and biotic factors [16,20,21]. Their usefulness for preventing food oxidation has also been evaluated, for example for meat products [16,19,22], dairy products and vegetable oils [19]. Their application for food packaging has also been investigated, with or without chitosan [16,18,23,24]. Olive leaf extracts combined with chitosan have been applied successfully to obtain films for nuts [25] and burgers [8] and applied as a coating agent to fruit [26,27], but not to burgers. However, to our knowledge, there is no information on the usefulness of combining them as a coating for burgers, despite the potential advantages of such a coating for extending their shelf-life. This study evaluates for the first time the addition of an olive leaf extract to a chitosan-based coating to improve pork burger preservation.

The aim of this study was to evaluate the effectiveness of an olive leaf extract added to a chitosan-based coating at delaying the deterioration of refrigerated pork burgers without food additives packaged under a 40% oxygen and 60% carbon dioxide modified atmosphere and to evaluate the interaction between the coating and storage time.

## 2. Materials and Methods

### 2.1. Biological Materials

Fresh pork was purchased from a local supermarket and ground (5 mm) with a meat mincer (Ramon, Vilassar de Dalt, Spain). The minced pork was formed into burgers (90 ± 5 g, 100 mm diameter) using a manual burger former (MH-100, Mainca, Granollers, Spain) without adding any other ingredients or additives to our food processing plant just before the experiment. Chitosan (deacetylation degree: 91.2%, purity: 91%; CAS number: 9012-76-4) and olive leaf extract powder (oleuropein content: 7.2%; purity: 95%; CAS Number 8060-29-5) were purchased from Guinama (Valencia, Spain).

### 2.2. Edible Coating Formulation and Application and Sampling

A procedure based on [28] was applied. Briefly, an aqueous solution of 1% (*w*/*v*) citric acid with 20 g of chitosan/Kg solution was stirred for 24 h to obtain a homogeneous solution, and 11.2 g glycerol/Kg was then added as a plasticizer agent to obtain the Chitosan solution. To obtain the Chitoex solution, the olive leaf extract was added to reach 1% *w*/*v*. The burgers were immersed in the coating solutions for 5 min and left to drip for 2 min.

The burgers were randomly allotted to each experimental treatment. Burgers without coating (Control), with Chitosan coating (Chitosan) and with Chitoex coating (Chitoex) were packaged individually in polypropylene (PP) trays (130 × 160 × 50 mm^3^, Sarabia Plastics, Alicante, Spain) sealed with a complex microperforated film (ACSAfilms, Valencia, Spain) with 100 μm pore diameter and consisting of 15 μm thick bioriented PP and 40 μm thick PP. Oxygen permeability was 680 ± 24 cm^3^/m^2^/24 h/ASTM, and carbon dioxide permeability was 2.5 ± 0.1 cm^3^/m^2^/24 h/ASTM. A ULMA Smart 500 sealer (Sevilla, Spain), a WITT gas blender and a WITT on-line gas analyzer (Witten, Germany) were used to introduce a mixture of gases in the packages consisting of 60% O_2_ + 40% CO_2_. The trays were kept under refrigeration (4 ± 1 °C) in the dark for 11 days, and they were monitored on days 0, 3, 6, and 11, when the experiment ended due to burger spoilage. Five replicates were performed, with a total of 50 packages (5 Control packages on day 0, and 3 coatings × 3 storage times × 5 replicates).

### 2.3. Headspace Gas Analysis, General Parameters and Microbial Counts

Oxygen and carbon dioxide were measured from 6 mL gas taken from each package through a patch just before opening, using a PBI Dansensor Checkpoint gas analyzer (Copenhagen, Denmark).

The weight loss was calculated after weighing the burgers before and after storage. Moisture was measured using the weights before and after desiccation at 102 °C for 24 h. pH was measured using a HANNA HI 99163 punction pHmeter (Woonsocket, RI, USA).

For microbial analyses, the samples (10 g) were taken aseptically and homogenized with 90 mL of peptone water (Pronadisa, Alcobendas, Madrid, Spain) in a Stomacher Lab Blender 400 (London, UK). A total of 0.1 mL of serial decimal dilutions in sterile peptone water were spread onto Plate Count Agar (Scharlau, Barcelona, Spain) for the aerobic psychrotrophic bacteria, which were incubated at 6.5 °C for 10 days [29], and onto MRS agar (Scharlau, Barcelona, Spain) for the lactic acid bacteria, which were incubated at 37 °C for 48 h.

### 2.4. Instrumental Color and Texture Measurement

A color measurement based on the CIE L*a*b* system [30] was performed using a Minolta Chroma Meter CR-300 (Osaka, Japan) with a D6 illuminant, a 10° angle, SCI and a measuring area of 30 mm^3^. The measurement was carried out on three different areas of the surface of each burger, and the averaged values for each burger were calculated and used in the statistical analysis. Each package was opened and left at room temperature (20 ± 2 °C) for approx. 30 min prior to the color measurements.

A TPA analysis [31] was performed after cutting the burgers in quarters, at room temperature, using the computer-assisted TA.XT Plus Texture Analyzer (ExponentStable Micro Systems, Godalming, UK). Compression was applied to the samples perpendicularly using a 5 kg load cell (Exponent Stable Micro Systems) and a 4mm diameter stainless cylinder probe (contact area: 28 mm^2^) (Stable MicroSystem, Godalming, UK), with a 5 mm compression distance and a crosshead speed of 1 mm/s, with two cycles. The TPA test was carried out in all the quarters of each burger, and the averaged values for each burger were calculated and used in the statistical analysis.

### 2.5. Lipid and Protein Oxidation

Lipid oxidation was measured via the thiobarbituric acid reactive substances (TBARs) method [32], using a standard curve of tetraethoxypropane (Sigma, St. Louis, MO, USA). Protein oxidation was assessed by estimating the thiol groups [33], using a standard curve of L-cysteine (Sigma, St. Louis, MO, USA).

### 2.6. Statistical Analysis

A one-way analysis of variance (ANOVA) was performed to check the effect of the storage time (days 0, 3, 6 and 11) on the Control samples (the other samples were not measured on day 0). A two-way (storage time and coating treatment) ANOVA with interaction was performed on the data from the stored samples. When the effect of any main factor was significant and there was not significant interaction, a post hoc Tukey test was performed to compare the means. For the parameters with significant interaction, an ANOVA simple effects test with the Sidak adjustment was performed. The analyses were carried out using the SPSS package (version 27, Chicago, IL, USA).

## 3. Results

Most of the parameters (all except weight loss, moisture and springiness) in the Control samples were affected by the storage time (day 0, 3, 6 and 11) according to the one-way ANOVA results (Table 1). For the stored samples (day 3, 6 and 11), the two-way ANOVA with interaction also revealed that the storage time affected most parameters (all except moisture), whereas the coating treatment had a weaker effect (11 out of 21 variables affected) (Table 1). However, the interaction was noticeable (13 out of 21 variables).

### 3.1. General Parameters and Microbial Counts

Table 1 shows the results from the ANOVA statistical analyses for the gas concentration, weight loss, moisture content, pH and the microbial counts of the burgers without coating (Control).

As for O_2_ and CO_2_, there was significant interaction between the storage time and the coating (Table 1). The effect of the coating treatment on them was not significant on day 3, whereas it was on day 6 and on day 11 (Table 2). Both the Chitosan and the Chitoex groups were significantly different from the Control group on days 6 and 11, resulting in higher O_2_ and lower CO_2_ concentrations, especially in the Chitoex samples (Table 2). These differences on day 6 match the differences in the counts for the aerobic psychrotrophic bacteria on day 6, although not on day 11. This suggests that those bacteria might not be the only cause for the differences. Therefore, both coatings were effective at limiting the drop in oxygen and the rise in carbon dioxide over storage, and the addition of leaf extract was also beneficial, although only on day 11.

With respect to the weight loss, the effect of the storage time was significant in the two-way ANOVA with interaction, although not strong enough to be significant in the Tukey test (Table 2), which is less powerful than the ANOVA, although the significance is small enough to suggest that a larger sample size might increase the statistical power and reveal a significant effect. The statistical significance of the moisture content from the one-way and two-way ANOVAs revealed that neither the storage time nor the coating treatment had any significant effect on it, nor did they have any interaction (Table 1).

Regarding pH, the highest values on day 3 (Table 2) showed that the coating, either with Chitosan or Chitoex, caused the lowest pH values on day 3, whereas on day 11 the effect was the opposite. In this respect, the coated samples experienced a rise in pH between day 6 and 11, whereas the Control samples did not. These results suggest that the coatings might prevent the slight rise in pH that occurred after 3-day storage in the Control burgers, whereas for longer storage the effect might be detrimental.

The results of the microbial counts revealed a significant effect of the coating treatment on days 3 and 6, but not on day 11, when all the counts were high (Table 2). Despite the effect on day 3, the Sidak test was not powerful enough to reveal any differences. On day 6 the coating, either with Chitosan or Chitoex, resulted in lower counts, and the addition of the olive leaf extract did not improve the outcome. Therefore, adding a chitosan coating was successful for controlling the aerobic psychrotrophic bacteria counts on day 6, but not on the other days, nor the lactic acid bacteria, with or without adding the olive leaf extract.

### 3.2. Instrumental Color and Texture

With respect to the instrumental color, the coating treatment had a slight effect (only *H*° affected), whereas the effect of the storage time was noticeable (all the variables affected), as it was the effect of the interaction (all affected except L*) (Table 1). a* and C* were significantly affected by the coating treatment only on day 11, with the Control samples being significantly different from the Chitosan and Chitoex samples, and with the lowest values in the Control group (Table 3). For b*, the effect of the coating treatment was significant on days 6 and 11 (Table 3); the Control group was significantly different from the Chitoex group on both sampling days, but with the opposite trend: on day 6 the Control group reached the lowest value, whereas on day 11 it reached the highest one (Table 3). With respect to *H*°, the effect of the coating treatment was significant on all the sampling days, the Control group was significantly different from the Chitoex group on all the sampling days, as well as different from the Chitosan group on days 3 and 11 (Table 3). Therefore, the coating affected the instrumental color, especially after 6-day storage, and the addition of the olive leaf extract had a significant yet modest effect.

As for the instrumental texture, hardness was significantly affected by the coating treatment only on day 11, with significant differences between the Control group and the two groups with coating, which reached the lowest values. With respect to gumminess and chewiness, they were affected by the coating treatment on day 6 and day 11, with the Control group having higher values than the Chitoex group on day 6, and higher values than both the Chitosan and Chitoex groups on day 11 (Table 4).

### 3.3. Lipid and Protein Oxidation

Lipid and protein oxidation were significantly affected by the coating treatment during the whole experiment (Table 5). For lipid oxidation, there were significant differences between the three groups, with the Control samples having the highest values and the Chitoex ones the lowest. The chitosan coating was effective in delaying lipid oxidation, and that the addition of the olive leaf extract increased the protective effect.

For protein oxidation, the Control samples were significantly different from the Chitosan samples only on day 6, and from the Chitoex ones on all the sampling days. The effect of the coatings was the opposite on day 3 (where they resulted in lower values than the Control group) and days 6 and 11 (where they resulted in higher values than the Control group).

## 4. Discussion

The results revealed that the coatings had a noticeable effect on the burgers, and that the effect depended on the storage time.

### 4.1. General Parameters and Microbial Counts

The concentration in O_2_ and CO_2_ in the package headspace might vary due to the microorganism metabolism, as well as due to their dissolution in the samples. The Chitosan coating and especially the Chitoex coating were useful to hinder the decrease in concentration in O_2_ and the increase in CO_2_ that happened over time. Therefore, not only chitosan, but also the olive leaf extract, was beneficial. The protective effect of both coatings against changes in O_2_ and CO_2_ might be related to the antimicrobial activity of chitosan [3] and the olive leaf extract [15,16,18]. However, the results of the microbial counts included in this study do not fully support this hypothesis, therefore, additional work is advisable to confirm the involvement of microorganisms in the changes in gas concentration.

With respect to the weight loss and moisture, the slight effect of the storage time on them (*p* = 0.006 for the former and not significant for the later) was likely due to the modified atmosphere packaging (MAP). This slight effect, with weight losses under 0.4% in all the groups, might be the cause of the lack of significant effect of the coating treatment, which was not useful to prevent those slight changes. Previous results on apple and strawberry revealed that chitosan and an olive leaf extract were effective at limiting weight loss [27]; however, it should be noted that the fruit was not packaged and suffered considerable weight losses. Therefore, although chitosan has a barrier effect [3], it might not be advantageous when using O_2_ and CO_2_ MAP.

Previous studies have reported that during storage in aerobic conditions, aerobic bacteria are involved in a rise in pH through protein decomposition [4], whereas in anaerobic conditions lactic acid bacteria (LAB) lead to a drop in pH through the production of lactic acid [34]. In our study, the presence of oxygen prevented anaerobic conditions, which might have prevented a decrease in pH. Although pH was affected by the storage time and the coating (Table 2), the values were stable (always in the 6.4–6.5 range) over time. The highest pH in the Control group on day 3 (when oxygen had not dropped yet) might be due to the presence of acid in the coating, as has been previously reported [4,35]. However, a previous study carried out on unpackaged chicken burgers (aerobic conditions) reported no effect when using a chitosan film with acetic acid on days 0 and 4, although after 20 days it prevented the rise in pH found in the Control samples [9].

With respect to the microbial counts, a significant effect of the Chitosan and Chitoex coatings on the aerobic psychrotrophic bacteria counts (with a 1.6–1.7 log cfu/g decrease) was only found on day 6 (Table 2). This decrease is in line with the 0.5–1.7 log cfu/g drop previously reported [36,37]. However, they had no effect on the LAB. The results for the effect of chitosan are roughly in line with other studies, which have reported that chitosan inhibited microbial growth in chicken burgers with chitosan films after 20 days of storage [9] and in chitosan-coated beef after 15 days [12], although in pork chops and chicken fillet the effect was also found during the early days of storage [35]. The differences between the studies might be related to the packaging and storage conditions, the initial counts, and the specific ingredients and additives. In our study, the lack of preservatives, such as sulfites, might have favored the increase in the microbial counts. In addition, the results show that the olive leaf extract did not increase the effect of chitosan. According to our results, the application of chitosan as a coating to pork burgers without additives and which are MAP-packaged did not have a marked beneficial effect on the microbial counts, and the addition of olive leaf extract did not provide any benefit from a microbial point of view.

General parameters such as weight loss, moisture content and pH, as well as the microbial counts, are indicators of quality loss and/or meat spoilage. Despite the marked effect of both Chitosan and Chitoex coatings on the headspace gases, they were not very effective at decreasing the already slight weight loss and moisture variations, nor were they very advantageous to the control of the microorganisms. The lack of a marked effect of the coating treatment on these parameters, therefore, indicates that the coating, with or without olive leaf extract, is not really effective at preventing the typical changes in burgers throughout refrigerated storage.

### 4.2. Instrumental Color and Texture

Color and texture are crucial for the quality of meat products. Not only does color influence, first, the consumers’ choice, but texture also influences repeated purchases [38]. Changes in color and texture are, therefore, relevant and critical for meat products. The results revealed that the coating treatment had a considerable effect on them.

With respect to the instrumental color, which is an indicator of meat freshness for consumers, the significant effect of the storage time on all the color parameters in the Control samples is in line with previous studies, which reported an increase in L* and a decrease in a* during the refrigerated storage of pork chops [35], and an increase in L* and a decrease in b* (a* was not reported) during the frozen storage of beef burgers [11]. It should be noted that for chicken meat (poorer in meat pigments), the effect of storage time on L* is the opposite, with a decrease over time [9,39].

The effect of the coating treatment depended to a great extent on the storage time (Table 1). Previous studies have not researched the effect of the storage time x chitosan-based coatings interaction. The marked effect of the coating treatment on the color parameters (all except L*) is in line with previous studies. With respect to L*, it has been shown that a chitosan coating caused an increase in L* [4,35]. Although Table 1 shows that the effect of the coating on L* was not significant, a similar trend was observed, with the highest values in the Chitosan group, and the relatively low significance (*p* = 0.066, Table 1) suggests that a more powerful statistical test or an increased sample size might reveal a significant effect.

Regarding a* and C*, the coating treatment affecting them only on day 11 (resulting in similar values for the Chitosan and Chitoex groups, and lower values in the Control group) indicates that the addition of the olive leaf extract did not improve the outcome and that the Chitosan coating had limited advantages, without being sufficient to prevent the changes linked to meat spoilage that occur during the first six days. On day 11, the results suggest that the Chitosan coating might be useful to prevent discoloration, although the drop in a* when compared to the initial values (they nearly halved) was still considerable. The beneficial effect of the Chitosan coating on a* is in line with previous results, which reported higher a* values in chitosan-coated pork [35,40]. This might be explained by the chelating effect of chitosan on transition metal ions [41].

As for b*, a significant but opposite effect of the coating treatment on days 6 and 11 was related to a larger drop in the values in the coated, rather than in the Control, samples after the first 6-day storage period (Table 3). This suggests that the coatings might be useful to prevent discoloration during the first six days but that later they might have a detrimental effect. In this regard, fluctuations in the effect of the chitosan coating on a* and b* have already been reported [4]. Regarding *H*°, the significant effect found on all the sampling days followed an opposite trend on days 3 and 6 (the coating treatment increased the values) compared to 11 (the treatment was successful at limiting the rise in *H*°) (Table 3), following a trend similar to b*, from which it was calculated.

Although texture is crucial for meat and meat products, previous information on the effect of chitosan-based coatings on texture is scarce and mainly related to the affective response [4]. Regarding hardness, the significant effect of the coating treatment only on day 11 (Table 4) suggests that it is beneficial only when storage is long enough to cause an increase in hardness. Both coating treatments, and especially the Chitoex one, were successful at maintaining hardness, gumminess, and chewiness close to the initial values, limiting the increase found in the Control group over storage. These results are in line with a previous study reporting the beneficial effect of a chitosan-based coating on the hedonic response to the texture of chicken burgers, which was attributed to less changes in the insoluble proteins of the muscle fibers and in the connective tissue [4].

A study reporting changes in burger texture (a decrease in juiciness and an increase in chewiness) over storage indicated that both traits are affected by protein oxidation and suggested that the texture changes might be related to the drop in the thiol concentration [22]. Our results for the thiol concentration are in line with some texture results (for example, in the Chitoex samples the thiols, hardness, gumminess and chewiness were not affected by the storage time). However, the trend is not as clear for the other groups, which suggests that there might be additional factors affecting the burger texture. The beneficial effect of the Chitosan coating might be explained by a decrease in the availability of oxygen to interact with meat proteins due to its barrier effect [3], decreasing protein oxidation (Table 5). The beneficial effect of the olive leaf extract on the texture might also be related to its antioxidant activity, which prevented lipid oxidation (Table 5). It should be noted that a detrimental effect of using chitosan-based films on the hedonic response to texture of chicken burgers was also reported [9]. These opposing results might be related to the differences in the use of chitosan (coating vs. film), as well to differences in the packaging parameters and burger characteristics.

### 4.3. Lipid and Protein Oxidation

Oxidation is one of the main factors limiting the shelf-life of meat and meat products. It is related not only to rancidity and discoloration [42] and, therefore, to the consumers’ perceptions, but also to some potential health benefits or drawbacks. The results showed that the effect of the coating treatment on oxidation was modulated by the storage time for both lipid and protein oxidation (Table 1).

Lipid oxidation was counteracted by the coating treatment, especially by the Chitoex coating, on all the sampling days. The Chitoex coating kept the values close to the initial ones even after six-day storage, markedly lower than the Control values (Table 5). The results for the Chitosan coating match previous results reporting significantly lower values in the chitosan group [9]. In this respect, it has been reported that chitosan decreases lipid oxidation in meat [11,12,35,39,40,43]. This protective effect of chitosan might be due to its O_2_ barrier effect (limiting the interaction between the burgers and the oxygen in the tray headspace); to lipid oxidation inhibition (the primary amino acid group of chitosan interacts with volatile aldehydes derived from the breakdown of oxidized fats [44]); and to the chelating effect of chitosan on transition metal ions [41]. In addition, our results showed the beneficial effect of including an olive leaf extract in the coating to delay lipid oxidation. It might be pointed out that no previous information on the effect of this natural extract combined with chitosan in a coating to prevent oxidation on meat products has been reported; however, a decrease in the MDA content has been reported in apples and strawberries when an olive leaf extract was included in a chitosan-based coating [27], and it has also been reported that this extract delays lipid oxidation [16,17,18].

Conversely to the effect on lipid oxidation, the effect of the coating treatment on protein oxidation was not as clearly beneficial. The Chitosan group only differed from the Control group on day 6, providing higher values for the thiol concentration. The Chitoex samples differed from the Control ones on days 3, 6 and 11, with lower values after 3-day storage but with an opposite trend after that. This apparently inconsistent effect over time was due to the fast drop in the thiol concentration for the Chitoex samples on day 3, which remained stable after then, whereas the values for the Control group did not decrease until day 6. The fast decrease caused by the olive extract does not match the results for lipid oxidation or the expected protective effect due to the antioxidant activity of the olive leaf extracts [16,18]. In this respect, a previous study on lamb burgers reported a decrease in the thiol concentration despite its beneficial effect to prevent lipid oxidation (TBARS) and carbonyl formation [22]. Its authors suggested that the decrease in thiols caused by the olive leaf extract was not related to oxidation reactions but to interactions between thiols and phenolic compounds with a catechol group [22], since it was found that they may form an adduct that is not susceptible to oxidation [45]. Further research is advisable to confirm this hypothesis and fully understand the effect of chitosan and olive leaf extracts on protein oxidation.

## 5. Conclusions

The effect of the chitosan-based coating was significant on most parameters, confirming the advantages previously reported on some parameters, such as some instrumental texture traits and lipid and protein oxidation. The addition of an olive leaf extract to a chitosan-based coating had no strong effect on some parameters, such as weight loss, moisture, pH, microbial counts, some instrumental texture variables and the color parameters. The lack of a noticeable inhibition of the microbial growth reveals that the treatment is not effective at extending the shelf-life greatly. However, the olive leaf extract was effective at preventing changes in texture (hardness, gumminess and chewiness) and decreasing lipid oxidation over storage, which indicates that it is beneficial to limit the changes in texture that occur over storage, as well as the oxidative reactions. These advantages indicate that a chitosan-based coating with a olive leaf extract could improve not only the consumers’ response to stored burgers, but also the oxidative status of the burgers, facilitating the production of healthier meat products.

## Figures and Tables

**Table 1:** Statistical significance from one-way (storage time) and two-way ANOVAs with interaction.

	One-Way	Two-Way (Stored Samples)		
	(Control Samples)	Time	Coating	Interaction
O_2_ *	<0.001	<0.001	<0.001	<0.001
CO_2_ *	<0.001	<0.001	<0.001	<0.001
Weight loss (%)	0.110	0.006	0.377	0.846
Moisture (%)	0.239	0.099	0.104	0.400
pH *	<0.001	<0.001	0.208	<0.001
Aerobic psychrotrophic bacteria *	<0.001	<0.001	0.005	<0.001
Lactic acid bacteria	<0.001	<0.001	0.894	0.109
L*	0.049	0.013	0.066	0.360
a* *	<0.001	<0.001	0.262	<0.001
b* *	<0.001	<0.001	0.153	0.008
C* *	<0.001	<0.001	0.550	0.028
*H*° *	<0.001	<0.001	0.029	<0.001
Hardness *	<0.001	<0.001	<0.001	<0.001
Adhesiveness	0.001	<0.001	0.278	0.188
Springiness	0.457	0.001	0.280	0.584
Cohesiveness	0.026	0.002	0.002	0.273
Gumminess *	<0.001	<0.001	<0.001	<0.001
Chewiness *	<0.001	<0.001	<0.001	<0.001
Resilience	<0.001	0.068	0.015	0.063
MDA *	<0.001	<0.001	<0.001	0.006
Thiols *	<0.001	<0.001	0.038	<0.001

* Due to the significant interaction, the simple effects test was performed, and the results are included in the next tables.

**Table 2:** Results (mean ± standard deviation) for headspace gas concentration, general parameters and microbial counts of burgers without coating (Control) and with a chitosan-based (Chitosan) or chitosan and olive leaf extract-based coating (Chitoex), packaged with a modified atmosphere and stored at 4 ± 1 °C.

		O_2_ (%)	CO_2_ (%)	Weight Loss (%)	Moisture (%)	pH	Aerobic Psychrotrophic Bacteria (log cfu/g)	Lactic Acid Bacteria (log cfu/g)
Day 0	Control	48.0 ± 2.1 ^1,2^	20.2 ± 0.9 ^1^	-	66.9 ± 0.4	6.4 ± 0.0 ^2^	3.5 ± 0.5 ^3^	5.3 ± 0.3 ^4^
Day 3	Control	50.7 ± 1.5 ^1^	19.2 ± 1.2 ^2^	0.1 ± 0.0	66.4 ± 1.6	6.5 ± 0.0 ^a,1^	6.5 ± 0.2 ^2^	6.4 ± 0.1 ^3^
	Chitosan	50.2 ± 1.6 ^1^	18.7 ± 0.9 ^2^	0.1 ± 0.0	68.3 ± 2.2	6.4 ± 0.0 ^b,2^	6.6 ± 0.2 ^3^	5.4 ± 0.8 ^2^
	Chitoex	51.4 ± 1.8 ^1^	18.4 ± 0.8 ^2^	0.2 ± 0.1	67.2 ± 3.1	6.4 ± 0.0 ^b^	7.3 ± 0.6 ^2^	6.6 ± 0.1
	*P_coating_*	*P_SE_*: 0.528	*P_SE_*: 0.414	*P_o_*: 0.009	*P_o_*: 0.448	*P_SE_* < 0.001	*P_SE_*: 0.043	*P_o_*: 0.440
Day 6	Control	45.4 ± 1.9 ^b,2^	20.8 ± 0.6 ^a,2^	0.3 ± 0.2	67.1 ± 2.2	6.4 ± 0.0 ^b,2^	8.9 ± 0.7 ^a,1^	7.1 ± 0.1 ^2^
	Chitosan	48.4 ± 1.8 ^a,1^	19.6 ± 1.0 ^b,2^	0.3 ± 0.2	65.9 ± 7.6	6.4 ± 0.0 ^b,3^	7.2 ± 0.3 ^b,2^	7.2 ± 0.3 ^1^
	Chitoex	49.0 ± 1.8 ^a,1^	19.2 ± 0.8 ^b,2^	0.3 ± 0.2	70.8 ± 0.7	6.4 ± 0.0 ^a^	7.3 ± 0.3 ^b,2^	7.0 ± 0.1
	*P_coating_*	*P_SE_*: 0.019	*P_SE_*: 0.019	*P_o_*: 0.993	*P_o_*: 0.249	*P_SE_* < 0.001	*P_SE_* < 0.001	*P_o_*: 0.553
Day 11	Control	3.6 ± 2.7 ^c,3^	57.2 ± 1.0 ^a,1^	0.2 ± 0.1	68.3 ± 1.2	6.4 ± 0.1 ^b,2^	8.5 ± 0.5 ^1^	7.8 ± 0.2 ^1^
	Chitosan	7.4 ± 2.1 ^b,2^	54.5 ± 1.9 ^b,1^	0.3 ± 0.2	69.8 ± 1.9	6.5 ± 0.0 ^a,1^	8.1 ± 0.2 ^1^	8.3 ± 0.9 ^1^
	Chitoex	14.2 ± 3.5 ^a,2^	49.6 ± 2.5 ^c,1^	0.4 ± 0.2	71.7 ± 4.4	6.5 ± 0.0 ^a^	8.4 ± 0.2 ^1^	7.5 ± 1.3
	*P_coating_*	*P_SE_* < 0.001	*P_SE_* < 0.001	*P_o_*: 0.442	*P_o_*: 0.212	*P_SE_* < 0.001	*P_SE_*: 0.359	*P_o_*: 0.612

*P_coating_*: statistical significance of the coating treatment on each sampling day from a one-way ANOVA (*P_o_*) when no significant interaction was found or from the ANOVA simple effects test (*P_SE_*) when there was interaction. Different superscript letters (a, b, c) within each variable and sampling day indicate significant differences at the *p* < 0.05 level in the Tukey test (when there was not significant interaction) or the Sidak test (when there was significant interaction). Different superscript numbers (1, 2, 3, 4) within each variable indicate significant differences between the storage times within the same coating treatment at the *p* < 0.05 level in the Tukey test.

**Table 3:** Results (mean ± standard deviation) for the instrumental color of burgers without coating (Control) and with a chitosan-based (Chitosan) or chitosan and olive leaf extract-based coating (Chitoex), packaged with a modified atmosphere and stored at 4 ± 1 °C.

		L*	a*	b*	C*	*H*°
Day 0	Control	50.9 ± 1.6 ^1,2^	20.3 ± 1.1 ^1^	12.4 ± 0.6 ^1^	23.8 ± 1.2 ^1^	31.3 ± 0.2 ^2^
Day 3	Control	50.0 ± 2.4 ^2^	19.0 ± 0.7 ^1^	11.6 ± 0.3 ^1^	22.3 ± 0.7 ^1^	31.3 ± 0.4 ^c,2^
	Chitosan	52.2 ± 1.7	18.1 ± 1.7 ^1^	11.6 ± 0.7 ^1^	21.5 ± 1.8 ^1^	32.7 ± 1.1 ^b,2^
	Chitoex	50.4 ± 1.5	17.2 ± 0.7 ^1^	12.1 ± 0.5 ^1^	21.1 ± 0.8 ^1^	35.1 ± 0.5 ^a^
	*P_coating_*	*P_o_*: 0.217	*P_SE_*: 0.077	*P_SE_*: 0.213	*P_SE_*: 0.311	*P_SE_* < 0.001
Day 6	Control	51.7 ± 1.7 ^1,2^	16.8 ± 1.0 ^2^	9.8 ± 0.6 ^b,2^	19.5 ± 1.2 ^2^	30.3 ± 0.3 ^b,2^
	Chitosan	52.9 ± 1.3	16.8 ± 0.8 ^1^	10.5 ± 0.9 ^ab,1,2^	19.9 ± 0.7 ^1^	32.0 ± 3.0 ^ab,2^
	Chitoex	51.7 ± 0.9	15.9 ± 1.5 ^1^	11.1 ± 0.6 ^a,2^	19.4 ± 1.4 ^2^	34.9 ± 2.2 ^a^
	*P_coating_*	*P_o_*: 0.298	*P_SE_*: 0.392	*P_SE_*: 0.047	*P_SE_*: 0.799	*P_SE_*: 0.017
Day 11	Control	53.6 ± 1.6 ^1^	9.0 ± 0.8 ^b,3^	9.9 ± 0.5 ^a,2^	13.4 ± 0.6 ^b,3^	47.7 ± 3.0 ^a,1^
	Chitosan	53.0 ± 1.2	11.7 ± 1.0 ^a,2^	9.4 ± 0.3 ^ab,2^	15.1 ± 0.8 ^a,2^	38.8 ± 2.5 ^b,1^
	Chitoex	51.6 ± 1.9	12.0 ± 0.5 ^a,2^	9.2 ± 0.2 ^b,3^	15.2 ± 0.3 ^a,3^	37.5 ± 1.7 ^b^
	*P_coating_*	*P_o_*: 0.178	*P_SE_* < 0.001	*P_SE_*: 0.038	*P_SE_*: 0.001	*P_SE_* < 0.001

*P_coating_*: statistical significance of the coating treatment on each sampling day from a one-way ANOVA (*P_o_*) when no significant interaction was found or from the ANOVA simple effects test (*P_SE_*) when there was interaction. Different superscript letters (a, b, c) within each variable and sampling day indicate significant differences at the *p* < 0.05 level in the Tukey test (when there was not significant interaction) or the Sidak test (when there was significant interaction). Different superscript numbers (1, 2, 3) within each variable indicate significant differences between the storage times within the same coating treatment at the *p* < 0.05 level in the Tukey test.

**Table 4:** Results (mean ± standard deviation) for the gas concentration, general parameters and microbial counts of burgers without coating (Control) and with a chitosan-based (Chitosan) or chitosan and olive leaf extract based-coating (Chitoex), packaged with a modified atmosphere and stored at 4 ± 1 °C.

		Hardness (N)	Adhesiveness (N × s)	Springiness (cm)	Cohesiveness	Gumminess (N)	Chewiness (N × cm)	Resilience
Day 0	Control	73.00 ± 4.96 ^2^	−14.31 ± 3.64 ^1^	0.89 ± 0.05	0.52 ± 0.02 ^1^	38.03 ± 1.33 ^1,2^	33.81 ± 1.95 ^2^	0.17 ± 0.01 ^1^
Day 3	Control	71.48 ± 1.71 ^2^	−18.90 ± 3.46 ^1^	0.91 ± 0.03	0.49 ± 0.01 ^2^	35.28 ± 2.43 ^2^	32.14 ± 2.72 ^2^	0.14 ± 0.01 ^2^
	Chitosan	77.27 ± 5.27	−16.32 ± 5.39 ^1^	0.87 ± 0.04 ^2^	0.47 ± 0.02	36.05 ± 1.88 ^2^	31.35 ± 1.21 ^3^	0.14 ± 0.02
	Chitoex	73.49 ± 8.39	−19.44 ± 9.41 ^1^	0.90 ± 0.04 ^1,2^	0.50 ± 0.02	36.29 ± 4.09	32.74 ± 3.91	0.15 ± 0.01
	*P_coating_*	*P_SE_*: 0.349	*P_o_*: 0.731	*P_o_*: 0.291	*P_o_*: 0.056	*P_SE_*: 0.855	*P_SE_*: 0.743	*P_o_*: 0.586
Day 6	Control	81.34 ± 7.72 ^2^	−22.24 ± 5.18 ^1^	0.91 ± 0.02	0.52 ± 0.01 ^1,2^	41.85 ± 3.76 ^a,2^	38.12 ± 3.25 ^a,2^	0.15 ± 0.01 ^1,2^
	Chitosan	85.91 ± 11.60	−17.60 ± 3.22 ^1^	0.89 ± 0.03 ^1,2^	0.47 ± 0.03	39.74 ± 3.64 ^ab,1,2^	35.17 ± 2.21 ^ab,2^	0.14 ± 0.01
	Chitoex	73.03 ± 7.51	−11.16 ± 2.32 ^1^	0.90 ± 0.03 ^2^	0.48 ± 0.02	35.23 ± 2.86 ^b^	31.64 ± 3.39 ^b^	0.15 ± 0.01
	*P_coating_*	*P_SE_*: 0.119	*P_o_*: 0.002	*P_o_*: 0.437	*P_o_*: 0.010	*P_SE_*: 0.029	*P_SE_*: 0.017	*P_o_*: 0.095
Day 11	Control	111.11 ± 5.06 ^a,1^	−33.64 ± 10.33 ^2^	0.93 ± 0.05	0.52 ± 0.02 ^1^	58.35 ± 3.26 ^a,1^	54.35 ± 5.64 ^a,1^	0.17 ± 0.01 ^1^
	Chitosan	87.16 ± 7.13 ^b^	−29.37 ± 5.74 ^2^	0.93 ± 0.02 ^1^	0.51 ± 0.03	43.83 ± 3.34 ^b,1^	40.79 ± 2.78 ^b,1^	0.15 ± 0.01
	Chitoex	76.72 ± 10.05 ^b^	−35.04 ± 10.23 ^2^	0.95 ± 0.02 ^1^	0.51 ± 0.03	39.15 ± 3.61 ^b^	37.11 ± 3.23 ^b^	0.14 ± 0.01
	*P_coating_*	*P_SE_* < 0.001	*P_o_*: 0.599	*P_o_*: 0.648	*P_o_*: 0.568	*P_SE_* < 0.001	*P_SE_*: <0.001	*P_o_*: 0.007

*P_coating_*: statistical significance of the coating treatment on each sampling day from a one-way ANOVA (*P_o_*) when no significant interaction was found or from the ANOVA simple effects test (*P_SE_*) when there was interaction. Different superscript letters (a, b) within each variable and sampling day indicate significant differences at the *p* < 0.05 level in the Tukey test (when there was not significant interaction) or the Sidak test (when there was significant interaction). Different superscript numbers (1, 2, 3) within each variable indicate significant differences between the storage times within the same coating treatment at the *p* < 0.05 level in the Tukey test.

**Table 5:** Results (mean ± standard deviation) for the lipid and protein oxidation of burgers without coating (Control) and with a chitosan-based (Chitosan) or chitosan and olive leaf extract-based coating (Chitoex), packaged with a modified atmosphere and stored at 4 ± 1 °C.

		MDA (mg/kg)	Thiol (nmol/mg Protein)
Day 0	Control	0.25 ± 0.11 ^3^	214.81 ± 7.75 ^a,1^
Day 3	Control	1.39 ± 0.27 ^a,2^	219.04 ± 26.12 ^a,1^
	Chitosan	0.83 ± 0.37 ^b^	204.82 ± 24.55 ^a,1^
	Chitoex	0.32 ± 0.09 ^c^	163.60 ± 7.22 ^b^
	*P_coating_*	*P_SE_* < 0.001	*P_SE_*: 0.004
Day 6	Control	2.38 ± 0.50 ^a,1^	119.52 ± 11.92 ^b,2^
	Chitosan	1.24 ± 0.19 ^b^	160.44 ± 16.51 ^a,2^
	Chitoex	0.34 ± 0.17 ^c^	160.15 ± 12.37 ^a^
	*P_coating_*	*P_SE_* < 0.001	*P_SE_*: 0.001
Day 11	Control	2.41 ± 0.26 ^a,1^	130.63 ± 3.53 ^b,2^
	Chitosan	1.26 ± 0.20 ^b^	144.45 ± 7.20 ^a,b,2^
	Chitoex	0.80 ± 0.28 ^c^	147.56 ± 14.12 ^a^
	*P_coating_*	*P_SE_* < 0.001	*P_SE_*: 0.032

*P_coating_*: statistical significance of the coating treatment on each sampling day from a one-way ANOVA (*P_o_*) when no significant interaction was found or from the ANOVA simple effects test (*P_SE_*) when there was interaction. Different superscript letters (a, b, c) within each variable and sampling day indicate significant differences at the *p* < 0.05 level in the Tukey test (when there was not significant interaction) or the Sidak test (when there was significant interaction). Different superscript numbers (1, 2, 3) within each variable indicate significant differences between the storage times within the same coating treatment at the *p* < 0.05 level in the Tukey test.

## Data Availability

The data supporting the findings of this study are available from the corresponding author upon reasonable request.
